# Effects of balance physical therapy with or without cognitive training in adults with cognitive and balance impairments : a systematic review

**DOI:** 10.1186/s11556-025-00383-w

**Published:** 2025-10-01

**Authors:** Gulnaz Magauina, Michalis Tsoukatos, Christos Nikitas, Sofia Papadopoulou, Dimitris Kikidis, Nattawan Utoomprurkporn, Patcharaorn Limkitisupasin, Doris-Eva Bamiou

**Affiliations:** 1 Faculty of medicine, Chulalongkorn University, Bangkok, Thailand; 2 National and Kapodistrian University of Athens, Athens, Greece; 3 University College London, London, United Kingdom

**Keywords:** Balance training, Balance exercises, Balance physiotherapy, Cognitive exercises, Cognitive training, Cognitive impairment, Cognitive disorders, Cognitive dysfunction, Mild cognitive impairment (MCI), Dementia, And alzheimer’s disease

## Abstract

**Background:**

Cognitive impairments, including MCI and dementia, significantly heighten fall risk due to motor dysfunction and balance deficits. Although physical activity is essential for dementia prevention, older adults often struggle with balance issues, fear of falling, and reduced mobility. This study investigated the impact of balance training, alone or combined with cognitive exercises, on functional balance and cognitive performance in individuals with cognitive impairments.

**Methods:**

A comprehensive literature search was conducted across three electronic databases to identify peer-reviewed studies written in English that examined the effects of balance-oriented physical therapy, either alone or in combination with cognitive training, on individuals with cognitive or concurrent cognitive and balance impairments. The outcomes of interest included balance and cognitive function. The risk of bias was evaluated independently by two reviewers using the ROB-1 tool. The effectiveness of the intervention was analyzed using RevMan software.

**Results:**

This systematic review found that stand-alone physical exercise significantly improved postural stability in 15 out of 24 studies and enhanced cognitive function in 5 out of 25 studies. Furthermore, the integration of cognitive training alongside physical exercise demonstrated additional benefits in improving balance and cognition in 7 out of 11 studies. These findings suggest that such interventions may be beneficial for older adults with cognitive impairments, warranting further research to establish definitive conclusions.

**Conclusion:**

This systematic review emphasizes the potential benefits of physical balance exercises, often combined with cognitive training, in improving balance, cognitive function, and certain aspects of quality of life among individuals with cognitive impairment.

**Supplementary Information:**

The online version contains supplementary material available at 10.1186/s11556-025-00383-w.

## Introduction

The established association between cognitive factors and gait control has been well-documented in the literature [1, 2]. Studies indicate that individuals experiencing gait difficulties face nearly double the risk of developing dementia (hazard ratio 1.96 [95% CI 1.30 to 2.96]) [3]. Moreover, individuals diagnosed with MCI are particularly vulnerable to falls due to gait abnormalities, diminished balance control, and reduced physical capacity [4]. Estimates suggest that the global prevalence of MCI ranges from 5.0 to 36.7% [5].

Recent evidence indicates that targeted exercise regimens and multifactorial interventions are promising in improving postural control, cognitive function, and fall incidence among individuals with MCI [6], potentially slowing the progression towards dementia [7]. Guidelines from organizations such as the National Institute for Health and Care Excellence (NICE) in the UK [8] and the American Physical Therapy Association [9] emphasize the importance of balance-focused physiotherapy, including both static and dynamic exercises, as a primary intervention for balance-related disorders. Reviews from Cochrane have also provided moderate to strong evidence supporting the benefits of balance rehabilitation in individuals with vestibular conditions [10] and those recovering from stroke [11].

Unfortunately, access to specialized balance rehabilitation services is limited or unavailable in many countries due to shortages of specialized professionals and healthcare resources to adequately address the needs of an aging population [12]. Additionally, prevalent home-based exercise programs often have a 50% attrition rate in follow-up [13]. These programs also have shortcomings, such as the absence of multisensory and cognitive components and a lack of focus on practical challenges, such as navigating crowded environments or complex settings like supermarkets [14, 15]. It is recommended that interventions should be tailored and individualized following thorough assessments and conducted under supervision to improve adherence [9].

However, the demand for these interventions exceeds the availability of proficient experts capable of delivering these services to middle-aged and older adults dealing with balance disorders and increased fall risks. To enhance adherence, interventions should be personalized based on comprehensive assessments and conducted under supervision when possible.

Given the increasing demand for specialized interventions, there is a growing need to evaluate their accessibility and effectiveness for middle-aged and older adults experiencing balance impairments and heightened fall risks.

## Methods

This systematic review was registered in the PROSPERO database (CRD42023406935) and conducted in accordance with the PRISMA 2020 guidelines [16] for systematic reviews and meta-analyses.

### Inclusion/exclusion criteria

#### Participants

This review included studies involving community-dwelling adults aged 18 and older diagnosed with cognitive impairment, including classifications such as mild cognitive impairment (MCI), based on the criteria used by each individual study. Eligible participants had Montreal Cognitive Assessment (MoCA) scores below 23, encompassing individuals with MCI, dementia, or Alzheimer’s disease. Studies involving children, adolescents, or nonhuman subjects were excluded.

#### Intervention

Balance Training programs (home or clinic based) aimed at improving functional balance (gait, stability, fall risk etc.) with or without cognitive training, low cost bedside exercises.

#### Comparison/control

The control group consisted of individuals with cognitive impairments who received standard care without balance training interventions.

#### Outcomes

The primary outcomes included measures of both balance and cognitive function. Any validated and reliable assessment tools were accepted without restrictions.

#### Type of studies

Only randomized controlled trials (RCTs) examining the effects of balance training with or without cognitive exercises in populations with cognitive impairments were included. Studies were considered RCTs if they reported randomized participant allocation.

#### Context

Research carried out in community-based settings, memory clinics, or nursing care facilities met the eligibility criteria. Geographical location was not a limiting factor.

### Search strategy

An extensive search was conducted across multiple databases to identify studies on balance training (including static and dynamic forms), with or without cognitive training, aiming to enhance both balance and cognitive status in individuals with cognitive disorders. PubMed, EMBASE, and Scopus were thoroughly searched using database-specific filters to identify relevant controlled trials. To account for differences in terminology, Boolean operators (OR/AND) and truncation (*) were applied to connect related terms within and across key concepts:

((“Dementia“[MeSH] OR “Cognitive Dysfunction“[MeSH] OR “Alzheimer Disease“[MeSH] OR dementia* OR alzheimer*) OR ((cognit*) AND (((impair*) OR dysfunct*) OR difficult* OR defect*)))

### AND

((balance OR postural balance [Mesh]) AND (exercise [Mesh] OR training OR rehab*))

Filters applied: Randomized Clinical Trials. The search included studies published up until May 2023 (S1 Appendix).

### Data extraction

**Fig. 1:**
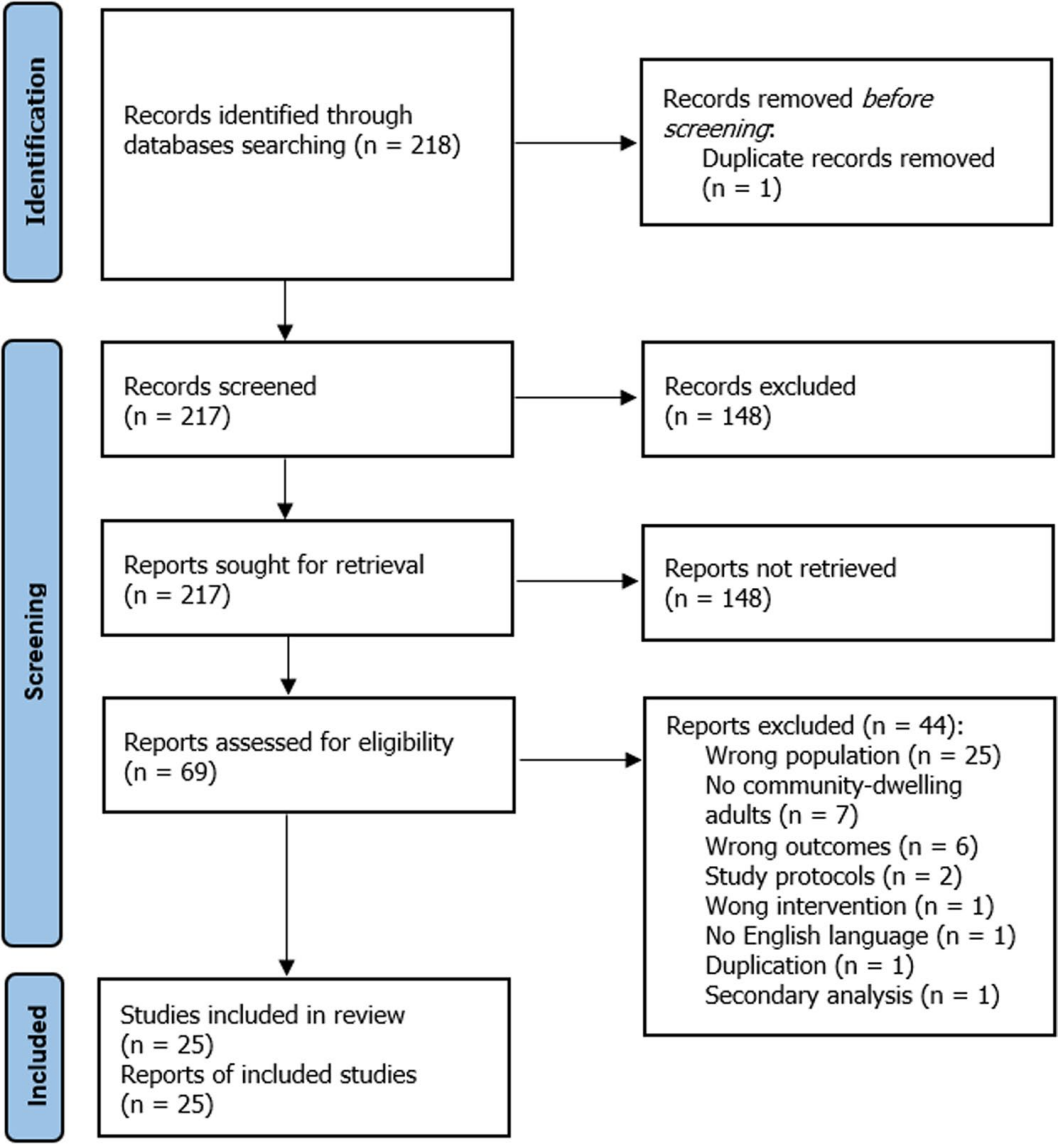
PRISMA flowchart

The search results were uploaded into Covidence to detect and eliminate duplicate studies [17]. Titles and abstracts retrieved from the databases were independently reviewed by the researchers. Articles deemed potentially eligible underwent an additional full-text evaluation by two reviewers (GM & MT) following the predetermined inclusion criteria. Any disagreements regarding study selection were resolved through discussion or consultation with a third reviewer (CN). Standardized data extraction forms, designed by the authors, were used to collect study details, including population characteristics, intervention and control conditions, outcome measures, key findings, and risk of bias assessment. Data extraction was performed separately by two reviewers (GM & SP), with discrepancies resolved through discussion. The selection process and the rationale for excluding studies are shown in a PRISMA 2020 flowchart (Fig. 1).

### Risk of bias assessment

The quality of the included studies was assessed independently by two reviewers (GM & MT), with disagreements resolved through discussion or consultation with a third reviewer (CN). The ROB-1 tool [18] was applied to assess bias risk in five primary areas, including randomization, allocation concealment, blinding, and selective reporting. Each area was classified as having a “Low,” “Unclear,” or “High” risk, with the overall bias risk being determined based on these ratings.

### Data analysis

The data collected encompassed essential details such as the study hypothesis, design, population, interventions, outcome measures, effects, and conclusions. Heterogeneity was assessed with the I² statistic (25%, 50%, and 75% indicating low, moderate, and high heterogeneity). As most pooled outcomes showed very high heterogeneity (I² >75%), a meta-analysis was not performed. Each article was reviewed, and its data and conclusions about the effects of balance interventions, with or without cognitive training, on adults with cognitive impairment were analyzed qualitatively. Data from the included studies were organized and summarized using Review Manager (RevMan, version 5.4.1; The Cochrane Collaboration) to assist with structured data presentation, including tables and figures.

## Results

### Study selection

Figure 1 presents the process flow for the Literature review based on PRISMA 2020 (S2 Appendix) guidelines [16]. Initially, 218 records were retrieved from database searches; after removing one duplicate, 148 studies were excluded based on their titles and abstracts. Sixty-nine articles underwent full-text screening, and 44 were excluded for reasons including incorrect study populations (*n* = 25), non-community-dwelling adults (*n* = 7), irrelevant outcomes (*n* = 6), study protocols (*n* = 2), wrong interventions (*n* = 1), non-English language (*n* = 1), duplication (*n* = 1), and secondary analysis (*n* = 1). In the end, 25 publications with a total of 1536 participants (mean age 74.53 ± 1.38 years) were included for qualitative analysis in the systematic review.

### Risk of bias in included studies

**Fig. 2:**
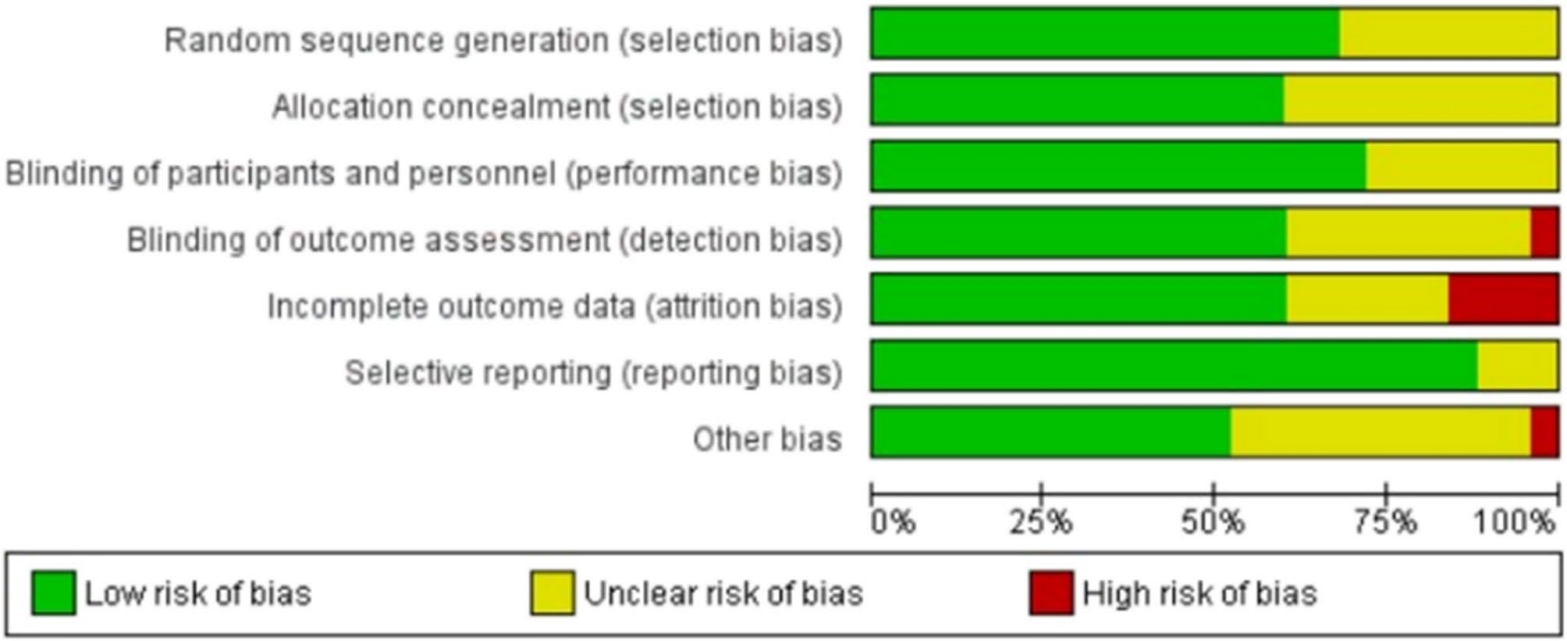
Risk of bias of the included studies

**Fig. 3:**
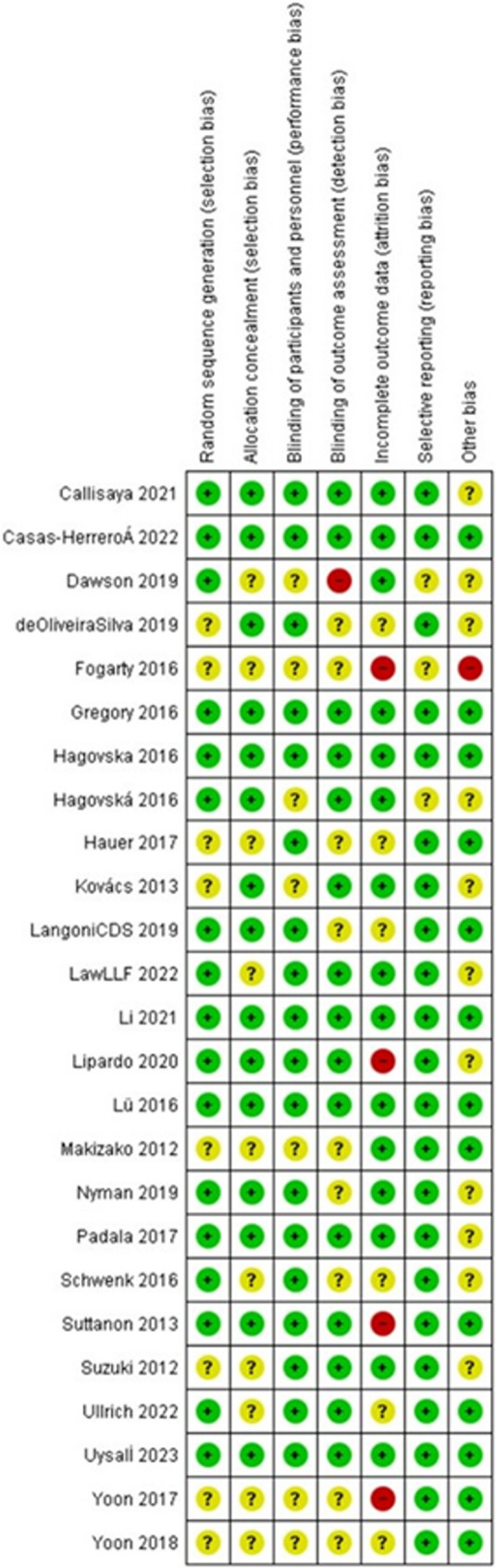
Risk of bias of fhe included studies

Risk of bias in the included studies is shown in Figs. 2 and 3. Among the included studies, seventeen seven studies [20–24, 27–29, 32, 34–39, 41, 42] were randomized by computer-generated numbers, and the remaining eight [19, 25, 26, 30, 31, 34, 40, 43] did not report the method of random sequence generation. Ten studies [20, 25, 26, 30, 32, 34, 40–43] did not report allocation concealment, and the rest [19, 21–24, 27–29, 31, 33, 35–39] all used a sealed envelope or box. Eighteen studies [19–24, 27–29, 32–38, 41, 43] blinded participants, and other seven studies [25, 26, 30, 31, 39, 40, 42] didn’t report participatn blinding. Blinding of outcome assessors was unclear in nine studies [19, 25, 26, 30, 32, 34, 35, 38, 40], one study [42] had no blinding, while outcome assessment was blinded in the remaining fifteen studies [20–24, 27–29, 31, 33, 36, 37, 39, 41, 43]. The collective kappa value for assessing the risk of bias in the included randomized controlled trials (RCTs) was 0.287, indicating a fair level of agreement between the two authors. To resolve this disagreement, the third author intervened and addressed the conflict in assessing the risk of bias in the studies.

### Study characteristics

Table 1 presents the characteristics and findings of 25 studies examining the effects of physical balance exercises, with or without cognitive training, in individuals with mild cognitive impairment, including amnestic mild cognitive impairment, dementia, Alzheimer’s disease, and cognitive frailty. Of these, ten studies combined balance and cognitive training [20, 25, 27–29, 33, 35–37, 39], while the remaining studies [19, 20, 22–24, 26, 30–32, 34, 38, 40–43] focused solely on balance training. The duration of physical training varied from 4 weeks to 12 months. All studies were conducted with community-dwelling older adults experiencing cognitive impairment. Examination of heterogeneity across frequently reported outcomes demonstrated substantial variability, with inconsistency levels very high for the Berg Balance Scale (BBS, I² = 78%), the Timed Up-and-Go Test (TUG, I² = 87%), and the Short Physical Performance Battery (SPPB, I² = 80%). Such heterogeneity limited comparability between studies and precluded a reliable meta-analysis, making a qualitative synthesis the most appropriate approach.

**Table 1:** Characteristics of included studies

Study(Year and location)	Study Design	Population Diagnosis	Sample size, *N* of participants in each group, Female (*n*,%).	Mean age	Intervention Group (IG)	Control Group (CG)	Duration of intervention
deOliveiraSilva, 2019, Brazil [19]	RCT	MCI AD	*N* = 46 MCI: IG = 7 (6, 85.71%) CG = 12 (5, 41.66%) AD: IG = 13 (5, 38.46%) CG = 14 (11, 78.57%)	MCI: IG = 71.85 ± 5.69 CG = 78.20 ± 5.26 AD: IG = 81.22 ± 8.88 CG = 77.54 ± 8.05	Multimodal physical training, including aerobic exercises, strength, balance, and stretching (60 min/session; 2 sessions/week)	Clinical follow-up without any physical training	12 weeks
LawLLF, 2022, China [20]	RCT	MCI	*N* = 145 FTE = 34 (23, 67.6%) ET = 37 (21, 56.8%) CT = 38 (25, 65.8%) WC = 36 (24, 66.7%)	FTE = 73.21 ± 7.27 ET = 77.35 ± 6.66 CT = 76.32 ± 7.21 WC = 74.14 ± 7.53	FTE group: received FTE training for 8 weeks, facilitated by a trained occupational therapist (12 sessions,60-min/session, 4–6/group) CT group: received an existing computer-based CT of attention, memory, executive function and visual perceptual function (12 sessions, 60-min/session, 4–6/group) ET group: exercises facilitated by an occupational therapist and an assistant for 8 weeks.(12 sessions,60 min/session, 4–6/group)	WC group: normal activities and exercise practice during the 8-weeks period	8 weeks
Lü, 2016, China [21]	RCT	MCI	*N* = 45 IG = 22 (16, 72.7%) CG = 23 (16, 69.6%)	IG = 69.00 ± 3.83 CG = 70.43 ± 5.53	Intervention group (DTG): Momentum-based dumbbell training (60 min/session, 3 sessions/week)	A regular lifestyle routine without starting any new exercise activities.	12 weeks
Casas-HerreroÁ, 2022, Spain [22]	RCT	MCI	*N* = 188 IG = 88 (63, 71.6%) CG = 100 (69, 69.0%)	IG = 84.2 ± 4.8 CG = 84.0 ± 4.8	Vivifrail multicomponent exercise programme (resistance/power, balance, flexibility and cardiovascular endurance exercises) (5 sessions/week)	Usual care	12 weeks
Padala, 2017, USA [23]	RCT	AD	*N* = 30 IG = 15 (5, 33.3%) CG = 15 (6, 40.0%)	IG = 72.1 ± 5.3 CG = 73.9 ± 7.1	Wii-Fit exercise program (yoga, strength training, aerobics, balance games) (30 min/session, 5 sessions/week)	Walking program (30 min/session, 5 sessions/week)	8 weeks
Suttanon, 2013, Thailand [24]	RCT	AD	*N* = 40 IG = 19 (13, 68.4%) CG = 21 (12, 57.1%)	IG = 83.42 ± 5.10 CG = 80.52 ± 6.01	The Otago Program home based exercise program 6 months	Education program (same number of home visits and phone calls)	24 weeks
Fogarty, 2016, UK, Canda [25]	RCT	MCI	*N* = 40 IG = 22 (12, 54.5%) CG = 18 (9, 50.0%)	IG = 71.55 ± 9.33 CG = 72.61 ± 5.78	Memory Intervention Program (MIP) and Taoist Tai Chi (TTC) training twice weekly for 90 min at a time for 10 weeks. (90 min/session, 2 sessions/week)	Memory Intervention Program (MIP) 8 sessions, with 6 sessions focusing on both education about lifestyle factors that impact memory and teaching of memory strategies and two follow-up sessions.	10 weeks
Yoon, 2018 Korea [26]	RCT	Cognitive frailty	*N* = 43 IG = 20 (14, 70.0%) CG = 23 (16, 69.6%)	IG = 73.82 ± 4.37 CG = 74.03 ± 4.27	A high-speed resistance training program. Independent exercise. (60 min/session, 3 sessions/week)	Routine daily activities and performed static and dynamic stretching (using elastic exercise band) (60 min/session, 2 sessions/week)	16 weeks
Callisaya, 2021 Australia [27]	RCT	MCI	*N* = 93 IG = 44 (27, 61.4%) CG = 49 (27, 55.1%)	IG = 72.9 ± 7.2 CG = 72.8 ± 6.9	Participants received an iPad with the StandingTall program. The program built to a total of 2 h of balance exercises per week, with cognitive dual-tasking exercises added in week 8. [40–2 h/week (from 40 min in weeks 1 and 2, to 120 min from week 9 onwards)]	Health information fact sheets via post on topics such as eyesight, diet, footwear and physical activity for the duration of the trial.	6 months
Uysalİ, 2023, Turkey [28]	RCT	MCI	*N* = 48 AG = 12 (2, 16.67%) DG = 12 (2, 16.67%) ADG = 12 (2, 16.67%) CG = 12 (2, 16.67%)	AG = 73.5 ± 3.21 DG = 74.08 ± 7.67 ADG = 73.25 ± 2.01 CG = 74.08 ± 7.82	Group 1: Aerobic exercise training combined with lower limb strengthening group (AG), (3 sessions/week) Group 2: Dual-task training combined with lower limb strengthening group (DG), (3 sessions/week) Group 3: Aerobic exercise training combined with dual-task training and lower limb strengthening group (ADG) (3 sessions/week)	Only lower limb strengthening group (CG). (3 sessions/week)	12 weeks
Li, 2021, USA [29]	RCT	MCI	*N* = 30 IG = 15 (9, 60.0%) CG = 15 (12, 80.0%)	IG = 76.13 ± 6.2 CG = 76.20 ± 6.3	Dual-task Tai Ji Quan training program based on Tai Ji Quan: Moving for Better Balance (60 min/session, 2 sessions/week)	Stretching exercises	24 weeks
Makizako, 2012, Japan [30]	RCT	aMCI	*N* = 50 IG = 25 (12, 48.0%) CG = 25 (11, 44.0%)	IG = 75.3 ± 7.5 CG = 76.8 ± 6.8	The six-month-long, multicomponent exercise program, with combinations of aerobic exercise, muscle strength training and postural balance retraining. (90 min/session, 2 sessions/week)	Education classes during the study period	24 weeks
Kovács, 2013, Hungary [31]	RCT	CI	*N* = 86 IG = 43 (36, 83.0%) CG = 43 (34, 79.0%)	IG = 76.39 ± 9.63 CG = 79.29 ± 12.67	The multimodal exercise program twice a week for 12 months	Usual care	12 months
Schwenk, 2016, USA [32]	RCT	aMCI	*N* = 22 IG = 12 (7, 58.3%) CG = 10 (5, 50.0%)	IG = 77.8 ± 6.9 CG = 79.0 ± 10.4	Sensor-based balance training program (45 min/session, 2 sessions/week)	No training	4 weeks
Hagovská, 2016, Slovak Republic [33]	RCT	MCI	*N* = 80 IG = 40 (18, 45.0%) CG = 40 (21, 52.0%)	IG = 68.0 ± 4.4 CG = 65.9 ± 6.2	Cogni-Plus, from SCHUHFRIED GmbH, Austria, 10 weeks (30 min/session, 7 sessions/week)	Dynamic balance training, 10 weeks (30 min/session, 7 sessions/week)	10 weeks
Hauer, 2017 Germany [34]	RCT	CI	*N* = 34 IG = 17 (11, 65.0%) CG = 17 (12, 71.0%)	IG = 81.4 ± 6.6 CG = 83.3 ± 5.7	Standardized 6-week home training.	Usual care	6 weeks
Nyman, 2019, UK [35]	RCT	Dementia	*N* = 85 IG = 42 (18, 43.0%) CG = 43 (16, 37.0%)	IG = 77.9 ± 8.3 CG = 78.2 ± 7.5	Tai Chi intervention: 3 components: (1) Tai Chi classes, (2) home-based Tai Chi practice, and (3) behaviour change techniques (90 min/session, 1 sessions/week)	Usual care	20 weeks
Lipardo, 2020, China [36]	RCT	MCI	*N* = 92 PACT = 23 (16, 70%) PT = 23 (22, 96%) CT = 23 (28, 78%) WG = 23 (17, 74%)	PACT = 67 ± 8.0 PT = 73 ± 7.0 CT = 68 ± 7.5 WG = 68 ± 8.5	Group 1: (PACT) physical and cognitive training. (60–90 min/session, 1–3 sessions/week) Group 2: (PT) physical training. (60–90 min/session, 1–3 sessions/week) Group 3: (CT) cognitive training. (60–90 min/session, 1–3 sessions/week)	Control: Group 4: (WG) waitlist group	12 weeks
Gregory, 2016, Canada [37]	RCT	MCI	*N* = 44 IG = 23 (15, 65.2%) CG = 21 (15, 71.4%)	IG = 72.6 ± 7.4 CG = 74.5 ± 7.0	(EDT) Group-based exercise + Dual Task training. (60–75 min/session, 2–3 sessions/week)	(EO) Exercise only. (60–75 min/session, 2–3 sessions/week)	26 weeks
LangoniCDS, 2019, Brazil [38]	RCT	MCI	*N* = 52 IG = 26 (20, 76.9%) CG = 26 (20, 76.9%)	IG = 72.6 ± 7.8 CG = 71.9 ± 7.9	Twice weekly sessions of group exercises, with volume and intensity regularly adjusted. (60 min/session, 2 sessions/week)	Usual life routine.	24 weeks
Hagovská, 2016, Slovak Republic [39]	RCT	MCI	*N* = 80 IG = 40 (18, 45%) CG = 40 (21, 52%)	IG = 68.2 ± 6.7 CG = 65.7 ± 5.6	CogniPlus training program from SHUFRIED GmbH Company in Austria: CogniPlus 20 training sessions, 2 sessions/week), physical training (30 min/session, 7 sessions/week)	Motor training (30 min/session, 7 sessions/week)	10 weeks
Yoon, 2017, Korea [40]	RCT	MCI	*N* = 30 HSPT = 14 (14, 100%) LSST = 9 (9, 100%) CON = 7 (7, 100%)	HSPT = 75.0 ± 3.46 LSST = 76.0 ± 3.94 CON = 78.0 ± 2.77	Group 1: (HSPT) Elastic bandbased high-speed power training (60 min/session, 2 sessions/week) Group 2: (LSST) Low-speed strength training (60 min/session, 2 sessions/week)	Group 3: (CON) Out balance and tone routines	12 weeks
Ullrich, 2022, Germany [41]	RCT	CI	*N* = 118 IG = 63 (48, 76.2%) CG = 55 (42, 76.4%)	IG = 82.2 ± 5.8 CG = 82.4 ± 6.2	Home-based training program (7 sessions/week)	A training manual	12 weeks
Dawson, 2019, USA [42]	RCT	Dementia	*N* = 23 IG = 13 (6, 46.2%) CG = 10 (7, 70%)	IG = 73.8 ± 8.5 CG = 74.0 ± 10.4	Moderate-intensity home-based functional exercise program, consisting of strength and balance exercises. (2 sessions/week)	Continuation of current levels of activity.	12 weeks
Suzuki, 2012, Japan, [43]	RCT	aMCI	*N* = 50 IG = 25 (12, 48%) CG = 25 (11, 44%)	IG = 75.3 ± 7.5 CG = 76.8 ± 6.8	Multicomponent exercise under the supervision of physiotherapists (90 min/session, 2 sessions/week)	Education control group	12 months

*AD* Alzheimer Disease, *ADG*Aerobic Exercise Training Combined with Dual-Task Training, *AG* Aerobic Exercise Group, *aMCI *Amnestic Mild Cognitive Impairment, *CG* Control Group, *CI* Cognitive Impairment, *CON* Control Group, *CT *Cognitive Training, *DG* Dual-Task Training Group, *DTG* Dumbbell Training Groupm, *ET* Exercise Training, *FTE* Functional Task Exercise, *HSPT* High-Speed Power Training, *IG* Intervention Group, *LSST* Low-Speed Strength Training, *MCI* Mild Cognitive Impairment, *PACT* Physical and Cognitive Training, *PT* Physical Training, *RCT* Randomized Controlled Trial, *WC* Wait-List Control

### Outcome measurements

The systematic review assessed outcomes in three domains, including balance, cognition, functional improvement, and quality of life.

Twenty-five RCTs examined the change in balance status using various outcome measures. These included: BBS [19, 21, 23, 25, 27, 29, 31, 32, 34, 36, 38, 40]; TUG [19, 20, 22, 24, 27, 28, 31, 33, 37, 38, 40]; SPPB [20, 23, 28, 32, 33, 36, 39]; ABC (Activities-specific Balance Confidence Scale) [19, 24, 28, 32, 35]; Step Test [22, 31]; Timed Chair Stand [25, 26]; mCTSIB (Modified Clinical Test of Sensory Interaction on Balance) [27, 33]; Limits of Stability (LOS) [29, 32]; WS (Walking Speed) [21, 23, 26, 30, 34, 36, 40].

The studies also measured the effect of the intervention on cognitive function using outcome measures such as: MMSE (Mini-Mental State Examination) [20, 21, 23, 25, 26, 29, 32, 35, 36, 38]; TMT-A (Trail Making Test Part A) [21, 24, 28, 31, 35]; TMT-B (Trail Making Test Part B) [19, 25, 31, 34]; MOCA (Montreal Cognitive Assessment) [22, 26, 27, 30, 33, 37, 38]; GDS (Geriatric Depression Scale) [20, 28, 31, 34]; NCSE (Naming, Connecting, Sensory, and Executive Function Test) [22, 30, 35]; ADAS-Cog (Alzheimer’s Disease Assessment Scale - Cognitive Subscale) [24, 29, 36]; HVLT (Hopkins Verbal Learning Test) [26, 31, 34]; MEC-Lobo (Lobo’s Mini-Exam for Cognitive Impairment) [25, 33]; Addenbrooke’s Cognitive Examination [27, 30, 36].

Additionally, nine studies examined the effect of treatment on various outcome measures such as: ADLQ (Activities of Daily Living Questionnaire) [21, 23, 27, 29, 35]; Lawton-IADL (Lawton Instrumental Activities of Daily Living Scale) [24, 31, 34]; HRQoL (Health-Related Quality of Life) [19, 22, 30, 32]; SF-12, SF-12 PCS, SF-12 MCS (Short Form Health Survey) [22, 28, 33, 36]; CST (Category Specific Therapy) [23, 32, 37]; Falls (Recording of Falls) [19, 24, 34, 38]; FROP-Com Falls Risk Score (Falls Risk Assessment) [20, 26, 33].

### Impact of physical rehabilitation on cognitive abilities in population with cognitive decline

A total of eight studies were reviewed to evaluate the effect of physical therapy on cognitive function in older adults with cognitive impairments (Table 2). All of these studies [21, 22, 28, 38, 40] specifically targeted individuals diagnosed with mild cognitive impairment (MCI).

**Table 2:** Effectiveness of physical training on cognitive function

Study(Year and location)	Study Design	Population Diagnosis	Mean age	Intervention parameters	Duration of intervention	Outcome Measures	Effect of intervention on Balance
Lü, 2016, China [21]	RCT	MCI	IG = 69.00 ± 3.83 CG = 70.43 ± 5.53	**IG**: Momentum-based dumbbell training (60 min/session, 3 sessions/week) **CG**: Usual care	12 weeks	Cognitive Tests: ADAS-Cog, TMT-B, DST-F	DTG had significantly improved ADAS – Cog subscale scores compared to the CG (5.02 points, *p* = 0.012)
Casas-HerreroÁ, 2022, Spain [22]	RCT	MCI	IG = 84.2 ± 4.8 CG = 84.0 ± 4.8	**IG**: Vivifrail multicomponent exercise programme (resistance/power, balance, flexibility and cardiovascular endurance exercises) (5 sessions/week) **CG**: Usual care	12 weeks	Cognitive Tests: MEC-Lobo, MOCA, GDS,	The intervention group showed improvements in the MOCA test after 3 months of exercise intervention (2.05 points; 95% CI 0.80, 3.28)
Uysalİ, 2023, Turkey [28]	RCT	MCI	AG = 73.5 ± 3.21 DG = 74.08 ± 7.67 ADG = 73.25 ± 2.01 CG = 74.08 ± 7.82	Group 1: Aerobic exercise training combined with lower limb strengthening group (AG), (3 sessions/week) Group 2: Dual-task training combined with lower limb strengthening group (DG), (3 sessions/week) Group 3: Aerobic exercise training combined with dual-task training and lower limb strengthening group (ADG) (3 sessions/week) **CG**: Usual care	12 weeks	Cognitive Tests: MMSE	In all three intervention groups, there was a signifcant improvement in cognitive status (*p* < 0.05).
LangoniCDS, 2019, Brazil [38]	RCT	MCI	IG = 72.6 ± 7.8 CG = 71.9 ± 7.9	**IG**: Twice weekly sessions of group exercises, with volume and intensity regularly adjusted. (60 min/session, 2 sessions/week) **CG**: Usual care	24 weeks	Cognitive Test: Geriatric Depression Scale-15, Addenbrooke’s Cognitive Examination Revised score	The intervention group showed significant improvement (*P* < 0.05) depressive symptoms (median punctuation (interquartile range) before: 4 (1.8–6); after: 2.5 (1–4))
Yoon, 2017, Korea [40]	RCT	MCI	HSPT = 75.0 ± 3.46 LSST = 76.0 ± 3.94 CON = 78.0 ± 2.77	Group 1: (HSPT) Elastic bandbased high-speed power training (60 min/session, 2 sessions/week) Group 2: (LSST) Low-speed strength training (60 min/session, 2 sessions/week) **CG**: Usual care	12 weeks	Cognitive Test: MMSE, MOCA.	In cognitive function, significant improvements in the MMSE and MOCA were seen in both the HSPT and LSST groups compared with the CON group.

*AD* Alzheimer Disease,*ADG*Aerobic Exercise Training Combined with Dual-Task Training, *AG* Aerobic Exercise Group,* aMCI* Amnestic Mild Cognitive Impairment, *CG* Control Group,* CI* Cognitive Impairment,* CON* Control Group,* CT* Cognitive Training,* DG* Dual-Task Training Group,* DTG* Dumbbell Training Group,*ET* Exercise Training,* FTE* Functional Task Exercise,* HSPT* High-Speed Power Training,* IG* Intervention Group,* LSST* Low-Speed Strength Training,* MCI* Mild Cognitive Impairment,* PACT* Physical and Cognitive Training, *PT* Physical Training,* RCT* Randomized Controlled Trial, *WC* Wait-List Control

MCI: The studies [21, 22, 28, 38, 40] provide evidence that various physical therapy interventions significantly enhance cognitive function in individuals with MCI. Momentum-based dumbbell training [21] led to improvements in ADAS-Cog scores (*F* = 6.95, *P* = 0.012), while the Vivifrail multicomponent exercise program [2] showed gains in MOCA scores (MD = 2.17; 95% CI: 0.61, 3.72; *P* < 0.05). Uysalİ et al. [28] found significant improvements in cognitive status (MMSE, d = 0.83, *P* < 0.05). Langoni et al. [38] reported reductions in depressive symptoms (GDS before: Median = 4; IQR 1.8–6. after: Median = 2.5; IQR 1–4, *P* < 0.05), and Yoon et al. [40] demonstrated significant improvements in MMSE and MOCA scores (d = 2.99 and d = 2.22, *P* < 0.05) through high-speed and low-speed strength training. Together, these findings highlight the beneficial effects of targeted exercise programs on cognitive health in MCI patients, suggesting that such interventions can play a vital role in mitigating or even reversing cognitive decline in MCI.

### Effect of physical rehabilitation on balance in population with cognitive impairments

Out of the 25 studies analyzed, 24 explored the effects of different physical therapy interventions on balance in individuals with cognitive impairments. Overall, 15 studies (see Table 3) revealed significant improvements in balance among patients with cognitive impairments who underwent physical therapy interventions alone.

**Table 3:** Effectiveness of physical training on balance

Study(Year and location)	Study Design	Population Diagnosis	Mean age	Intervention parameters (Intervention and Control Groups)	Duration of intervention	Outcome Measures	Effect of intervention on Balance
deOliveiraSilva, 2019, Brazil [19]	RCT	MCI AD	MCI: IG = 71.85 ± 5.69 CG = 78.20 ± 5.26 AD: IG = 81.22 ± 8.88 CG = 77.54 ± 8.05	**IG**: Multimodal physical training, including aerobic exercises, strength, balance, and stretching (60 min/session; 2 sessions/week) **CG**: Usual care	12 weeks	Mobilty Tests: ST_8UG, CoV_8UG, DT_8UG, DTC_8UG	Improvement in balance was observed in the IG compared to the CG, with a significant difference in the simple task mobility test (ΔCG: −0.18 ± 0.53; ΔIG: −1.05 ± 0.57; *P* = 0.03).
Lü, 2016, China [21]	RCT	MCI	IG = 69.00 ± 3.83 CG = 70.43 ± 5.53	**IG**: Momentum-based dumbbell training (60 min/session, 3 sessions/week) **CG**: Usual care	12 weeks	Mobilty Tests: TUG, FR, ABC	Significant improvement in balance was noted in the IG as compared to the CG (TUG test: 0.81 s; p-value = 0.043).
Casas-HerreroÁ, 2022, Spain [22]	RCT	MCI	IG = 84.2 ± 4.8 CG = 84.0 ± 4.8	**IG**: Vivifrail multicomponent exercise programme (resistance/power, balance, flexibility and cardiovascular endurance exercises) (5 sessions/week) **CG**: Usual care	12 weeks	Mobilty Tests: SPPB	Significant improvement in balance was observed in the IG, with a mean increase of 0.86 points (95% CI 0.32, 1.41; *P* < 0.01) after 1 month and 1.40 points (95% CI 0.82, 1.98; *P* < 0.001) after 3 months.
Padala, 2017, USA [23]	RCT	AD	IG = 72.1 ± 5.3 CG = 73.9 ± 7.1	**IG**: Wii-Fit exercise program (yoga, strength training, aerobics, balance games) (30 min/session, 5 sessions/week) **CG**: Walking program	8 weeks	Mobilty Tests: BBS, ABC scale, FES,	The IG showed significantly greater improvements in balance compared to the CG, with notable differences in BBS (Δ = 4.8 [3.3–6.2], *p* < 0.001), ABC scale (Δ = 6.5 [3.6–9.4], *p* < 0.001), and FES (Δ = − 4.8 [–7.6 to − 2.0], *p* = 0.002).
Suttanon, 2013, Thailand [24]	RCT	AD	IG = 83.42 ± 5.10 CG = 80.52 ± 6.01	**IG**: The Otago Program home based exercise program 6 months **CG**: Usual care	24 weeks	Mobilty Tests: Falls, FROP-Com falls risk score, FR, Step Test, Timed Chair Stand, TUG, mCTSIB, Limits of stability (LOS), Walking, Step quick turn, Sit to stand	Significant improvement in Functional Reach (*P* = 0.002) in the IG compared to the CG.
Yoon, 2018 Korea [26]	RCT	Cognitive frailty	IG = 73.82 ± 4.37 CG = 74.03 ± 4.27	**IG**: A high-speed resistance training program. Independent exercise. (60 min/session, 3 sessions/week) **CG**: Usual care	16 weeks	Mobilty Tests: SPPB, TUG,	Significant improvements in balance were observed in the IG with better performance in SPPB and TUG (both *p* < 0.05).
Uysalİ, 2023, Turkey [28]	RCT	MCI	AG = 73.5 ± 3.21 DG = 74.08 ± 7.67 ADG = 73.25 ± 2.01 CG = 74.08 ± 7.82	Group 1: Aerobic exercise training combined with lower limb strengthening group (AG), (3 sessions/week) Group 2: Dual-task training combined with lower limb strengthening group (DG), (3 sessions/week) Group 3: Aerobic exercise training combined with dual-task training and lower limb strengthening group (ADG) (3 sessions/week)	12 weeks	Mobilty Tests: TUG, ABC, SLST	In all three intervention groups, Significant improvement in balance was observed in all intervention groups (AG, DG, ADG) with the most notable improvements in DG and ADG (*p* < 0.05).
Kovács, 2013, Hungary [31]	RCT	CI	IG = 76.39 ± 9.63 CG = 79.29 ± 12.67	**IG**: The multimodal exercise program twice a week for 12 months **CG**: Usual care	12 months	Mobilty Tests: POMA-B, POMA-G, POMA-T, TUG, falls.	Significant improvement in balance-related items of the POMA-B scale was observed in the IG at both 6 and 12 months (*P* < 0.0001 and *P* = 0.002, respectively).
Schwenk, 2016, USA [32]	RCT	aMCI	IG = 77.8 ± 6.9 CG = 79.0 ± 10.4	**IG**: Sensor-based balance training program (45 min/session, 2 sessions/week) **CG**: Usual care	4 weeks	Mobilty Tests: Gait speed, eyes open (EO), and eyes closed (EC). Anterior-posterior (AP, cm) sway, medial-lateral (ML, cm) sway, and total sway area (cm2) of the center of mass (CoM), Short-FES-I,	Significant reduction in sway (eyes open) in the IG compared to the CG (*p* = 0.041).
Hauer, 2017 Germany [34]	RCT	CI	IG = 81.4 ± 6.6 CG = 83.3 ± 5.7	**IG**: Standardized 6-week home training. **CG**: Usual care	6 weeks	Mobilty Tests: SPPB, POMA test, APAFOP, Sit-to-Stand (STS)	Significant improvements in SPPB (total score: *p* = 0.012; chair rise: *p* = 0.007; balance: *p* = 0.066), reduced gait and balance deficits in POMA (total score: *p* = 0.006; balance: *p* = 0.034; gait: *p* = 0.019), and increased physical activity (APAFOP; *p* = 0.05) in the IG compared to the CG.
Nyman, 2019, UK [35]	RCT	Dementia	IG = 77.9 ± 8.3 CG = 78.2 ± 7.5	**IG**: Tai Chi intervention: 3 components: (1) Tai Chi classes, (2) home-based Tai Chi practice, and (3) behaviour change techniques (90 min/session, 1 sessions/week) **CG**: No treatment	20 weeks	Mobilty Tests: TUG, BBS, Icon-FES, Falls	Significant improvement in TUG and BBS scores in the IG compared to the no-treatment group (WMD = 1.04, 95% CI: 0.67, 1.41 for TUG; WMD = 2.86, 95% CI: 1.91, 3.81 for BBS).
LangoniCDS, 2019, Brazil [38]	RCT	MCI	IG = 72.6 ± 7.8 CG = 71.9 ± 7.9	**IG**: Twice weekly sessions of group exercises, with volume and intensity regularly adjusted. (60 min/session, 2 sessions/week) **CG**: Usual care	24 weeks	Mobilty Tests: BBS, TUG	The IG showed significant improvement in balance, with a mean increase in BBS score from 53 ± 3 to 55.1 ± 1.1 points (*P* < 0.05).
Yoon, 2017, Korea [40]	RCT	MCI	HSPT = 75.0 ± 3.46 LSST = 76.0 ± 3.94 CON = 78.0 ± 2.77	Group 1: (HSPT) Elastic bandbased high-speed power training (60 min/session, 2 sessions/week) Group 2: (LSST) Low-speed strength training (60 min/session, 2 sessions/week) **CG**: Usual care	12 weeks	Mobilty Tests: SPPB, TUG.	Significant improvement in physical function was observed in both HSPT and LSST groups compared to the CG, with increased SPPB scores in the intervention groups.
Ullrich, 2022, Germany [41]	RCT	CI	IG = 82.2 ± 5.8 CG = 82.4 ± 6.2	**IG**: Home-based training program (7 sessions/week), a CI-specific, autonomous, home-based strength, balance, and walking training **CG**: Usual care	12 weeks	Mobilty Tests: SPPB, TUG, LSA-CI, Short FES-I	Significant improvement in physical function was observed in both HSPT and LSST groups compared to the CG, with increased SPPB scores in the intervention groups.
Dawson, 2019, USA [42]	RCT	Dementia	IG = 73.8 ± 8.5 CG = 74.0 ± 10.4	**IG**: Moderate-intensity home-based functional exercise program, consisting of strength and balance exercises. (2 sessions/week) **CG**: Usual care	12 weeks	Mobilty Tests: The 30-second chair stand test, m-BBS, The 8-ft walk test,	Significant improvement in balance was observed in the IG compared to the CG, with better performance in balance and gait speed tests (t = 4.1, *P* = 0.001 for balance; t = 2.6, *P* = 0.02 for gait speed).

*AD*Alzheimer Disease, *ADG* Aerobic Exercise Training Combined with Dual-Task Training, *AG* Aerobic Exercise Group, *aMCI* Amnestic Mild Cognitive Impairment, *CG* Control Group, *CI* Cognitive Impairment, *CON* Control Grou, *CT* Cognitive Training,* DG* Dual-Task Training Group,* DTG* Dumbbell Training Group, *ET* Exercise Training, *FTE* Functional Task Exercise, *HSPT* High-Speed Power Training, *IG* Intervention Group, *LSST* Low-Speed Strength Training,* MCI* Mild Cognitive Impairment,* PACT* Physical and Cognitive Training, *PT * Physical Training, *RCT* Randomized Controlled Trial, *WC* Wait-List Control

Eleven studies [20–22, 28, 29, 33, 36–40] targeted individuals with MCI, three studies focused on those with general cognitive impairment [31, 34, 41], two studies addressed patients with Alzheimer’s Disease [23, 24], two studies involved patients with dementia [35, 42], one study examined patients with amnestic MCI (aMCI) [32], one study included both MCI and Alzheimer’s Disease patients [19], and one study concentrated on individuals with cognitive frailty.

MCI + AD: The study by de Oliveira Silva et al. [19] revealed that a 12-week multimodal physical training program, incorporating aerobic exercises, strength training, balance work, and stretching, resulted in notable improvements in simple task mobility in patients with MCI and AD, compared to a control group (Δ control group: −0.18 ± 0.53; Δ exercise group: −1.05 ± 0.57; *P* = 0.03).

MCI Lü et al. [21] demonstrated that momentum-based dumbbell training significantly enhanced balance, reducing TUG test times by 0.81 s (*p* = 0.043). Casas-Herrero et al. [22] observed substantial improvements in SPPB scores with the Vivifrail multicomponent exercise program, showing increases of 0.86 points (*P* < 0.01) after one month and 1.40 points (*P* < 0.001) after three months. Uysalİ et al. [28] observed significant improvements in cognitive status, balance, mobility, and other physical health measures across three groups— aerobic exercise, dual-task training, and a combination of both— with the combination group showing the most significant gains (*P* < 0.05). Langoni et al. [38] demonstrated that bi-weekly group exercise sessions led to significant improvements in balance (*P* < 0.05), with BBS scores increasing from 53 ± 3 to 55.1 ± 1.1 points, and TUG test times decreasing from 10.7 ± 2.9 s to 8.3 ± 2.0 s. Yoon [40] reported that both high-speed power training (d = 1.27, *P* < 0.05) and low-speed strength training (d = 1.10, *P* < 0.05) led to significant improvements in SPPB scores when compared to the control group.

Cognitive Impairment: The research by Kovács et al. [31], Hauer et al. [34], and Ullrich et al. [41] underscores the positive impact of structured physical training programs on enhancing balance and mobility in older adults with cognitive impairment. Kovács’s study demonstrated sustained improvements in balance, with POMA-B scores increasing from a median of 10 (IQR 7–14) at 6 months to 11 (IQR 8–14) at 12 months (*P* < 0.01). Meanwhile, interventions by Hauer (d = 0.23, *P* = 0.01) and Ullrich (between-group MD = 1.9 points; 95% CI: 1.0-2.8; *P* < 0.001) significantly enhanced functional performance, as reflected in improved SPPB scores. These findings highlight the benefits of regular, structured physical exercise programs tailored to individuals with cognitive impairment.

AD: The studies by Padala et al. [23] and Suttanon et al. [24] demonstrate significant improvements in balance and mobility among Alzheimer’s Disease (AD) patients through targeted exercise programs. Padala’s use of a Wii-Fit exercise program significantly enhanced balance and mobility as evidenced by improvements in BBS (MD = 4.8 points; 95% CI: 3.3–6.2, *P* < 0.001), Activities-specific Balance Confidence Scale (ABC scale) (MD = 6.5 point; 95% CI: 3.6–9.4, *P* < 0.001), and FES scores (MD=–4.8 points; 95% CI:–7.6 to − 2.0, *P* = 0.002), while Suttanon’s implementation of the Otago Program significantly increased Functional Reach (MD = 4.18 cm; 95% CI: 1.57 − 6.79, *P* = 0.002), highlighting the efficacy of tailored, structured exercise interventions in this population.

Dementia: The studies by Nyman et al. [35] and Dawson et al. [42] demonstrate significant enhancements in balance and mobility for individuals with dementia through structured exercise programs. Nyman’s Tai Chi intervention had a significantly lower rate of falls (RR = 0.35; 95% CI: 0.15–0.81, *P* = 0.015), while Dawson’s moderate-intensity home-based program not only improved balance (m-BBS: B = 4.04, t = 4.13, *P* = 0.001) but also resulted in better fast gait speed (B = 0.32, t = 2.61 *P* = 0.02) among participants, underlining the effectiveness of exercise in enhancing functional abilities in dementia patients.

aMCI In a study by Schwenk et al. [32], a sensor-based balance training program administered over four weeks showed a significant reduction in sway with eyes open (d = 0.224, *P* = 0.041) among patients with amnestic Mild Cognitive Impairment (aMCI). However, Makizako et al. [30] reported that the multicomponent exercise program did not yield statistically significant improvement in dual-task performances with balance demands (F_1,45_ = 3.3, *P* = 0.07).

Cognitive frailty Yoon et al. [26] found that a high-speed resistance training program significantly enhanced physical function in individuals with cognitive frailty. Over 16 weeks, with 60-minute sessions three times a week, the intervention resulted in significant improvements in the SPPB (Mean = 10.85 ± 1.60), TUG test (Mean = 9.26 s ± 2.03), and gait speed (Mean = 5.34 s ± 0.81), all showing statistical significance (*P* < 0.05).

In total, eleven studies [19, 21–24, 26, 28, 35, 38, 40, 42] documented significant improvements after physical therapy, addressing various facets of mobility, and balance improvement in patients with MCI, AD, dementia, and cognitive frailty.

### Effect of dual task exercises on balance and cognitive

Eleven studies [20, 25, 27–30, 33, 36, 37, 39, 43] investigated the effect of dual task exercises on balance and cognitive demands (Table 4). The range of exercises was from 8 to 24 week. Nine studies [20, 25, 27–29, 34, 36, 37, 39] focused on patients with mild cognitive impairment (MCI), while two studies [30, 43] focused on patients with aMCI.

**Table 4:** Physical training with cognitive training

Study(Year and location)	Study Design	Population Diagnosis	Intervention Group (IG)	Duration of intervention	Ouctomes	Effect of intervention
LawLLF, 2022, China [20]	RCT	MCI	FTE group: received FTE training for 8 weeks, facilitated by a trained occupational therapist (12 sessions,60-min/session, 4–6/group)	8 weeks	**Balance Outcome**: CST, BBS	Functional task exercise group improved balance (Berg Balance Scale) at Post intervention 5-month follow-up (Mean = 53.5 (3.67) 95% CI (− 1.60 to − 0.43)) (p-value = 0.039), and Chair Stand Test Mean 14.56 (5.59) 95% CI (− 4.21 to − 1.84) (p-value 0.000).
Fogarty, 2016, UK, Canda [25]	RCT	MCI	Memory Intervention Program (MIP) and Taoist Tai Chi (TTC) training twice weekly for 90 min at a time for 10 weeks. (90 min/session, 2 sessions/week)	10 weeks	**Cognitive Outcome**: HVLT, Digit Span and Digit Symbol from the Wechsler Adult Intelligence Scale–III, TMT-A, TMT-B, RBMT–II, TEA, MAC-SR, **Balance Outcome**: RAPA, Gait velocity, stride time, and stride time variability, CTSIB “	TTC exercise did not specifically improve cognition or physical mobility. There was no significant change over time for the MIP + TTC group compared with the MIP-alone group on any of the gait variables, on any of the dual-task cost variables, or in the amount of sway on any of the balance measures.
Callisaya, 2021 Australia [27]	RCT	MCI	Participants received an iPad with the StandingTall program. The program built to a total of 2 h of balance exercises per week, with cognitive dual-tasking exercises added in week 8. [40–2 h/week (from 40 min in weeks 1 and 2, to 120 min from week 9 onwards)]	6 months	**Cognitive Outcome**: TMT-A, TMT-B, Victoria Stroop task, COWAT, Digital Symbol Coding tests and the Hopkins Verbal Learning Test, **Balance Outcome**: Gait speed, Dual-task gait, FICSIT-4, ABC, Falls.	No significant differences between the methods on balance and cognition.
Uysalİ, 2023, Turkey [28]	RCT	MCI	Group 1: Aerobic exercise training combined with lower limb strengthening group (AG), (3 sessions/week) Group 2: Dual-task training combined with lower limb strengthening group (DG), (3 sessions/week) Group 3: Aerobic exercise training combined with dual-task training and lower limb strengthening group (ADG) (3 sessions/week)	12 weeks	**Cognitive Outcome**: MMSE **Balance Outcome**: TUG, ABC, SLST”	The most remarkable change was observed in the ADG on cognitive status, mobility and physical performance parameters (*p* < 0.05). In addition, the most signifcant improvement in balance parameters was recorded both in the DG and ADG (*p* < 0.05). Besides, the highest increase in functional exercise capacity was detected both in the AG and ADG (*p* < 0.05)
Li, 2021, USA [29]	RCT	MCI	Dual-task Tai Ji Quan training program based on Tai Ji Quan: Moving for Better Balance (60 min/session, 2 sessions/week)	24 weeks	**Balance Outcome**: Falls, 4-Stage Balance Test, 30-second chair stands, TUG,	The Tai Ji Quan program improved balance compared to the Stretching Group.
Makizako, 2012, Japan [30]	RCT	aMCI	The six-month-long, multicomponent exercise program, with combinations of aerobic exercise, muscle strength training and postural balance retraining. (90 min/session, 2 sessions/week)	24 weeks	**Balance Outcome**: WS, OLS, RT, Dual-task costs (DTC balance and cognitive demand)	The improvement effects on dual-task performances with both balance and cognitive demands were not statistically significant: reaction time with balance demand (*P* = 0.07), and cognitive demand (*P* = 0.12).
Hagovská, 2016, Slovak Republic [33]	RCT	MCI	Cogni-Plus, from SCHUHFRIED GmbH, Austria, 10 weeks (30 min/session, 7 sessions/week)	10 weeks	**Cognitive Outcome**: MMSE, Addenbrooke’s Cognitive Examination, **Balance Outcome**: BESTest, TUG, POMA test, Falls”	“There were significant differences between these two groups recorded in the assessment of several cognitive functions by Addenbrooke’s cognitive examination (p < 0.05–0.0001) in favor of the experimental group. The assessment of postural reactions and the total score of the BESTest were in favor of the experimental group (p < 0.05–0.0001)”
Lipardo, 2020, China [36]	RCT	MCI	Group 1: (PACT) physical and cognitive training. (60–90 min/session, 1–3 sessions/week) Group 2: (PT) physical training. (60–90 min/session, 1–3 sessions/week) Group 3: (CT) cognitive training. (60–90 min/session, 1–3 sessions/week)	12 weeks	**Balance Outcome**: TUG, The 10-Meter Walk Test, The 10-Meter Walk Test, Physiological Profile Assessment–Short Form, Falls rate	No significant difference was observed across time and groups on fall incidence rate at 12 weeks (*P* = 0.152) and at 36 weeks (*P* = 0.954). The groups did not statistically differ in other measures except for a significant improvement in dynamic balance based on Timed Up and Go Test in the combined physical and cognitive training group (9.0 s with *P* = 0.001) and in the cognitive training alone group (8.6 s with *P* = 0.012) compared to waitlist group (11.1 s) at 36 weeks.
Gregory, 2016, Canada [37]	RCT	MCI	(EDT) Group-based exercise + Dual Task training. (60–75 min/session, 2–3 sessions/week)	26 weeks	**Balance Outcome**: Gait	At 26 weeks, the EDT group demonstrated increased dual-task (DT) gait velocity [difference between groups in mean change from baseline (95% CI): 0.29 m/s (0.16–0.43), *p* < 0.001], DT step length [5.72 cm (2.19–9.24), *p* = 0.002], when compared to the EO group.
Hagovská, 2016, Slovak Republic [39]	RCT	MCI	CogniPlus training program from SHUFRIED GmbH Company in Austria: CogniPlus 20 training sessions, 2 sessions/week), physical training (30 min/session, 7 sessions/week)	10 weeks	**Cognitive Outcome**: MMSE, TMT-A, The Nine Hole Peg Test. **Balance Outcome**: BESTest, TUG, TUG DT with dual tasking. “	The cognitive-motor training performed for 10 weeks confirmed more significant relationships between balance control, cognitive functions, gait speed, and activities of daily living, when compared with motor intervention alone.
Suzuki, 2012, Japan, [43]	RCT	aMCI	Multicomponent exercise under the supervision of physiotherapists (90 min/session, 2 sessions/week)	12 months	**Cognitive Outcome**: MMSE, WMSLM I and II, WMS-LM, letter verbal fluency test (LVFT), category verbal fluency test: (CVFT), SCWT-I, SCWT-III	Improvements of cognitive function following multicomponent exercise were superior at treatment end (group × time interactions for the mini-mental state examination (*P* = 0.04), logical memory of immediate recall (*P* = 0.03), and letter verbal fluency test (*P* = 0.02)). The logical memory of delayed recall, digit symbol coding, and Stroop color word test showed main effects of time, although there were no group × time interactions.

*AD* Alzheimer Disease,*ADG* Aerobic Exercise Training Combined with Dual-Task Training,* AG* Aerobic Exercise Group,* aMCI *Amnestic Mild Cognitive Impairment, *CG* Control Group, *CI* Cognitive Impairment, *CON* Control Group,* CT* Cognitive Training, *DG* Dual-Task Training Group, *DTG* Dumbbell Training Group,*ET* Exercise Training,* FTE* Functional Task Exercise,* HSPT* High-Speed Power Training,* IG* Intervention Group,* LSST* Low-Speed Strength Training,* MCI* Mild Cognitive Impairment, *PACT* Physical and Cognitive Training,* PT* Physical Training, *RCT* Randomized Controlled Trial, *WC* Wait-List Control

MCI: In a study by Law et al. [20], an 8-week Functional Task Exercise (FTE) program significantly improved participants’ executive function (ADLQ: *P* = 0.044; d = 0.72; Lawton-IADL: *P* < 0.001; d = 1.01), problem-solving abilities (*P* < 0.001; d = 0.89), and physical performance (CST: *P* = 0.05; d = 0.59), with these positive effects sustained at the 5-month follow-up, including improvements in mental health-related quality of life. Lü et al. [21] reported significant improvements after 12 weeks of momentum-based dumbbell training (DTG), including a 5.02-point reduction in ADAS-Cog subscale scores (*P* = 0.012), enhanced functional mobility as shown by faster TMT-B performance (− 33.32 s, *P* < 0.001) and higher DST scores (+ 0.41 points, *P* = 0.025), along with improved balance. Casas-HerreroÁ et al. [22] observed increases in SPPB scores (MD = 1.40; 95% CI: 0.82–1.98; *P* < 0.001) and improvements in the MOCA test (MD = 2.17; 95% CI: 0.61–3.72; *P* = 0.014) with a 12-week Vivifrail multicomponent exercise program. Uysalİ et al. [28] conducted a 12-week exercise intervention study, finding notable improvements in cognitive function (MMSE MD(± sd) = − 2 ± 0, *P* < 0.001), balance (SPPB MD(± sd) = − 1.92 ± 0.29, *P* < 0.001), and overall quality of life (WHOQOL-OLD MD(± sd) = − 2,17 ± 2,82, *P* < 0.001), particularly in the group that incorporated a combination of aerobic exercise, dual-task training, and lower body strengthening (ADG). Hagovská et al. [33, 39] found significant cognitive and postural benefits with 10 weeks of dynamic balance (BESTest: d = 0.31, *r* = 0.15, *P* = 0.03) and cognitive training (Addenbrooke’s Cognitive Examination: d = 0.48, *r* = 0.23, *P* = 0.03). LangoniCDS et al. [38] reported significant improvements following 24 weeks of group exercise. Balance improved as indicated by an increase in BBS scores from 53 ± 3 to 55.1 ± 1.1 points (*P* < 0.05), mobility improved with a reduction in TUG times from 10.7 ± 2.9 to 8.3 ± 2.0 s, and depressive symptoms decreased, as shown by a reduction in GDS scores from a median of 4 (IQR 1.8–6) to 2.5 (IQR 1–4). Yoon et al. [40] found that a 12-week intervention with high-speed power training (HSPT) and low-speed strength training (LSST) led to significant improvements in cognitive function, as measured by the MMSE (20.76% improvement, d = 2.99 with HSPT; 13.91% improvement, d = 1.29 with LSST; *P* < 0.05 for both), and in physical performance, as measured by the SPPB (32.55% improvement, d = 1.27 with HSPT; 20.27% improvement, d = 1.10 with LSST; *P* < 0.05 for both).

aMCI: Makizako et al. [30] conducted a six-month multicomponent exercise program and found no significant impact on dual-task performance (DRT: F_1,45_=3.3 ms, *P* = 0.07) in individuals with aMCI. In contrast, Suzuki et al. [43] examined the effects of a 12-month multicomponent exercise program led by physiotherapists on cognitive function in those with aMCI. The results demonstrated considerable improvements in cognitive function, with significant group × time interactions observed for the MMSE (MD = − 0.47, 95%CI:−1.75 − 0.81; *P* = 0.04), Logical Memory Immediate Recall (MD = 4.62, 95% CI:2.19 − 7.05; *P* = 0.03), and the Letter Verbal Fluency Test (MD = 2.99, 95% CI:0.69 − 5.30; *P* = 0.02) at the program’s conclusion.

Overall, seven studies evaluated [20, 28, 29, 33, 37, 39, 43] its effectiveness of dual task trainings in improving the physical and cognitive aspect in people with cognitive impairments. These findings underscore the positive impact of diverse exercise interventions with cognitive training on cognitive function and balance in older adults.

### Functional improvements and quality of life

Nine studies assessed the impact of physical therapy on older adults with cognitive impairment. Law et al. [20] reported significant improvements in functional status (ADLQ: *P* = 0.002; d = 0.79) and physical performance (CST: *P* = 0.008; d = 0.95) in the FTE group. Suttanon [24] found that the Otago Program reduced falls risk (β=−2.58, 95%CI: −4.49 to − 0.66; *P* = 0.008) and improved Limits of stability (β=−0.57, 95%CI: −1.04 to − 0.11; *P* = 0.016). Uysal et al. [28] observed notable improvements in all intervention groups, with the greatest gains seen in the group combining aerobic exercise, dual-task training, and lower limb strengthening (ADG). Li [29] observed no significant difference in fall rates (IRR = 0.58; 95%CI: 0.32 to 1.03) between participants who practiced Tai Ji Quan and those who performed stretching exercises. Kovács [31] also reported no significant differences in falls (RR = 0.77; 95%CI: 0.33 to 1.49) between groups. Schwenk [32] noted a reduction in fear of falling (d = 0.302, *P* = 0.015) in the sensor-based balance training group. Lipardo et al. [36] reported no significant differences in fall incidence rates between groups at both 12 weeks (*P* = 0.152) and 36 weeks (*P* = 0.954). Langoni et al. [38] reported a significant improvement in mobility within the intervention group, with TUG times decreasing from 10.7 ± 2.9 s to 8.3 ± 2.0 s. Yoon et al. [40] demonstrated significant increases in muscle strength in the HSPT and LSST groups. Ullrich et al. [41] demonstrated a significant improvement in falls efficacy in the home-based training group compared to the control group, as measured by the Short FES-I (β = −2.00, 95%CI: −3.48 to − 0.53, *P* = 0.008). Overall, the systematic review indicates that physical therapy interventions generally improve various aspects of older adults with cognitive impairment, including functional status, physical performance, mobility, muscle strength, and falls efficacy. However, the effectiveness of fall prevention specifically varies, suggesting the need for tailored and individualized approaches (Table 5).

**Table 5:** Summary of outcome measures and statistical significance in included RCTs

Outcome Measure	No. of Studies	Studies with Significant Difference	Studies with No Significant Difference
Balance Status			
BBS (Berg Balance Scale)	12	7 [19, 23, 25, 27, 31, 34, 36]	5 [21, 29, 32, 38, 40]
TUG (Timed Up-and-Go Test)	11	6 [19, 22, 24, 27, 28, 38]	5 [20, 31, 33, 37, 40]
SPPB (Short Physical Performance Battery)	7	4 [20, 23, 28, 34]	3 [32, 36, 39]
ABC (Activities-specific Balance Confidence Scale)	5	3 [19, 28, 32]	2 [24, 35]
Step Test	2	1 [22]	1 [31]
Timed Chair Stand	2	1 [25]	1 [26]
mCTSIB (Modified Clinical Test of Sensory Interaction on Balance)	2	1 [27]	1 [33]
Limits of Stability (LOS)	2	1 [29]	1 [32]
WS (Walking Speed)	7	4 [21, 23, 34, 36]	3 [26, 30, 40]
Cognitive Function			
MMSE (Mini-Mental State Examination)	10	6 [21, 23, 29, 32, 35, 38]	4 [20, 25, 26, 36]
TMT-A (Trail Making Test Part A)	5	3 [21, 24, 31]	2 [28, 35]
TMT-B (Trail Making Test Part B)	4	2 [20, 25]	2 [31, 34]
MOCA (Montreal Cognitive Assessment)	7	4 [22, 26, 27, 37]	3 [30, 33, 38]
GDS (Geriatric Depression Scale)	4	2 [20, 34]	2 [28, 31]
NCSE (Naming, Connecting, Sensory, and Executive Function Test)	3	2 [22, 30]	1 [35]
ADAS-Cog (Alzheimer’s Disease Assessment Scale - Cognitive Subscale)	3	2 [24, 36]	1 [29]
HVLT (Hopkins Verbal Learning Test)	3	2 [26, 34]	1 [31]
MEC-Lobo (Lobo’s Mini-Exam for Cognitive Impairment)	2	1 [33]	1 [25]
Addenbrooke’s Cognitive Examination	3	2 [27, 36]	1 [30]
Other Outcomes			
ADLQ (Activities of Daily Living Questionnaire)	5	3 [21, 23, 29]	2 [27, 35]
Lawton-IADL (Lawton Instrumental Activities of Daily Living Scale)	3	2 [24, 34]	1 [31]
HRQoL (Health-Related Quality of Life)	4	2 [19, 30]	2 [22, 32]
SF-12, SF-12 PCS, SF-12 MCS (Short Form Health Survey)	4	2 [28, 36]	2 [22, 33]
CST (Category Specific Therapy)	3	1 [23]	2 [32, 37]
Falls (Recording of Falls)	4	2 [24, 34]	2 [19, 38]
FROP-Com Falls Risk Score (Falls Risk Assessment)	3	1 [26]	2 [20, 33]

## Discussion

### Summary of findings

The objective of this study was to explore the effects of balance training, both with and without cognitive training, on cognitive abilities, postural stability, and other outcomes in individuals with cognitive impairment. We reviewed 25 studies and evaluated their quality using the ROB-1 tool [18]. Out of these, eight studies specifically addressed the effects of balance training on cognitive function in older adults, with seven focusing on mild cognitive impairment (MCI) and one examining amnestic MCI (aMCI). The interventions demonstrated significant improvements in cognitive abilities, including executive functions, cognitive scores (ADAS-Cog, MOCA), and reductions in depressive symptoms, highlighting the efficacy of physical therapy in enhancing cognitive health and potentially managing cognitive decline in MCI patients. The study on aMCI [42] also indicated significant cognitive improvements, suggesting the benefits of physical therapy across different cognitive conditions.

Out of the 25 studies analyzed, 24 explored the impact of physical therapy on balance among other outcomes, with 15 studies demonstrating significant improvements. These studies showed significant improvements in balance metrics such as the TUG and BBS across various subgroups, including those with MCI, AD, dementia, and cognitive frailty. Most studies have reported positive effects on balance.

Among the studies that examined the impact of dual-task exercises, 7 out of 11 showed positive outcomes. These exercises, which integrate physical and cognitive tasks, proved particularly effective at boosting both cognitive and physical abilities, suggesting their value in holistic cognitive rehabilitation approaches.

The research also demonstrated gains in wider functional capabilities and quality of life. This included improvements in daily problem-solving, mental health-related quality of life, mobility, muscle strength, and effectiveness in preventing falls, all of which are essential for maintaining the independence and overall well-being of individuals with cognitive impairments.

The systematic review indicates that physical therapy interventions, including balance and cognitive training, significantly improve cognitive functions, postural control, and other outcomes in patients with various forms of cognitive impairment.

In a previous systematic review by Kiper et al. [44], it was found that combining physical and cognitive training significantly improved balance compared to motor-only training (SMD 0.56; 95% CI 0.07 to 1.06; I2 = 59%; 160 participants). Additionally, the combined training group showed a significant improvement in mobility compared to no intervention (MD − 1.80; 95% CI − 2.70 to − 0.90; I2 = 0%; 81 participants). Ali et al. [45] reported similar results, where dual-task training led to improvements across various cognitive domains: global cognitive function (SMD = 0.24, *P* = 0.002), memory (SMD = 0.28, *P* = 0.001), executive function (SMD = 0.35, *P* < 0.001), attention (SMD = −0.19, *P* = 0.1), gait speed (SMD = 0.26, *P* = 0.007), dual-task cost (SMD = 0.56, *P* < 0.001), and balance (SMD = 0.36, *P* = 0.004). Gkotzamanis et al. [46] indicated that the impact of physical activity on cognitive function among individuals with varying cognitive statuses suggests a potential protective role of physical activity against cognitive decline before the onset of dementia. However, its effects on those already affected by dementia remain debatable.

### Strengths and limitations of this study

The primary strength of our review lies in the robust evidence derived from exclusively including RCTs, specifically focusing on older adults with cognitive impairment living in the community. Our study offers a comprehensive analysis of multiple outcomes—balance, cognitive function, functional improvements, and quality of life—providing a well-rounded understanding of the impact of balance training interventions. By including both single-task and dual-task (balance plus cognitive) training programs, we offer nuanced insights into effective rehabilitation strategies tailored to this vulnerable population. A significant advantage of this review is the comprehensive and transparent assessment of risk of bias across all included RCTs, as illustrated in Figs. 2 and 3. Most studies reported adequate randomization methods and blinding of participants or outcome assessors, which enhances the internal validity of our findings. Furthermore, any discrepancies in risk of bias judgments were resolved through consensus with a third reviewer, ensuring methodological rigor and reliability in the quality appraisal process. Additionally, detailed reporting of intervention characteristics—such as frequency, duration, and exercise type—enhances the clinical applicability and reproducibility of our findings. Our adherence to PRISMA 2020 guidelines further strengthens the transparency and methodological quality of this review. Importantly, by evaluating a broad range of outcome domains—including cognitive function, balance, mobility, muscle strength, and quality of life—our review captures the multifaceted benefits of balance training, underscoring its potential to improve the overall well-being and functional independence of individuals with mild cognitive impairment.

This systematic review has several limitations. One significant limitation is the absence of a formal meta-analysis. Although many studies reported similar outcome measures, such as the BBS, TUG, and SPPB, the high variability in intervention types, training parameters, and study populations—particularly among individuals with cognitive impairment—restricted our ability to statistically pool the data with adequate confidence. Previous reviews, such as those by Lesinski et al. [47], successfully conducted meta-analyses and established dose-response relationships for balance training in healthy older adults. However, their findings were based on more homogeneous populations and well-defined training protocols. In contrast, the studies we included often lacked detailed reporting on training volume and intensity, which are essential for synthesizing dose-response effects.

Additionally, as noted by Draborg et al. [48], many original studies do not contextualize their results within previous systematic reviews, our review reflects a broader challenge in the field where the accumulation of comparable, high-quality data remains limited. Furthermore, our literature search was confined to English-language publications, potentially excluding relevant studies amid the expanding research on MCI globally. Future studies should concentrate on targeted interventions addressing cognitive issues in older adults, with the goal of slowing or preventing the progression of cognitive decline in MCI, dementia, and Alzheimer’s disease. Moreover, further research is needed to determine the optimal intensity, duration, frequency, and structure of balance and dual-task training programs to achieve sustained functional and cognitive benefits, as emphasized by Lesinski et al. [47].

Another limitation of our review is that, while several included studies reported statistically significant improvements in cognitive and balance outcomes, we did not assess whether these changes were clinically meaningful. We did not utilize a distribution-based approach to derive minimal clinically important differences (MCIDs) from baseline values, as recommended by Watt et al. [49].

## Conclusion

In conclusion, this analysis of 25 studies highlights the significant impact of balance training—whether or not it includes cognitive components—on improving balance, cognitive function, and overall functional outcomes in individuals with cognitive impairment. Most studies reported marked improvements in balance, including gait and performance-based measures such as the Activities-specific Balance Confidence (ABC) scale and the Timed Up and Go (TUG) test. Programs that incorporated aerobic exercise, dual-task training, and lower limb strengthening were particularly effective. Notably, even interventions lacking explicit cognitive components resulted in improvements in cognitive function, including executive functioning and global cognition. Functional outcomes, such as muscle strength, mobility, fall risk, and fear of falling, also showed consistent improvements. A systematic risk of bias assessment was conducted, with most studies demonstrating acceptable methodological quality; any disagreements were resolved by a third reviewer, enhancing the reliability of our findings. These results underscore the importance of individualized, multifaceted balance training programs in addressing the complex needs of older adults with cognitive impairment, ultimately supporting their quality of life and functional independence.

## Supplementary Information

Supplementary Material 1.

Supplementary Material 2.

## Data Availability

All data generated or analyzed during this study were extracted from previously published articles. Additional information is available from the corresponding author upon reasonable request.

## Abbreviations

ABC: Activities-specific Balance Confidence Scale
BBS: Berg Balance Scale
CI: Confidence Interval
CRD: Centre for Reviews and Dissemination
GDS: Geriatric Depression Scale
ICD-10: International Classification of Diseases, 10th Revision
LOS: Limits of Stability
MCI: Mild Cognitive Impairment
MMSE: Mini-Mental State Examination
MoCA: Montreal Cognitive Assessment
mCTSIB: Modified Clinical Test of Sensory Interaction on Balance
NICE: National Institute for Health and Care Excellence
PRISMA: Preferred Reporting Items for Systematic Reviews and Meta-Analyses
PROSPERO: International Prospective Register of Systematic Reviews
RCT: Randomized Controlled Trial
RevMan: Review Manager (software)
ROB-1: Risk of Bias Tool, Version 1
SPPB: Short Physical Performance Battery
TMT-A: Trail Making Test Part A
TMT-B: Trail Making Test Part B
TUG: Timed Up-and-Go Test
UK: United Kingdom
WS: Walking Speed
