# Supplementary material for: Effects of balance physical therapy with or without cognitive training in adults with cognitive and balance impairments : a systematic review
Source: Eur Rev Aging Phys Act. 2025 Oct 1;22:16. doi: 10.1186/s11556-025-00383-w (PMC12487321; doi:10.1186/s11556-025-00383-w)
Supplement: Supplementary file 1 — Supplementary Material 1. [file 11556_2025_383_MOESM1_ESM.pdf]

Appendix 1 Detailed search strategies for each database search terminology and results

**PubMed**		
Terms	((("Dementia"[MeSH] OR "Cognitive Dysfunction"[MeSH] OR dementia*) OR ((cognit*) AND (((impair*) OR dysfunct*) OR difficult* OR defect*))) AND ((balance AND (((impair*) OR dysfunct*) OR difficult* OR defect*)) OR accidental fall[Mesh]))	
	AND	
	((balance OR postural balance [Mesh] OR vestibular) AND (exercise [Mesh] OR training OR rehab* OR program))	
Total		215 papers
**Scopus**		
Terms	TITLE-ABS-KEY ("balance" OR "postural balance") AND ("training" OR "exercise")	
	AND	
	("dementia" OR "alzheimer" OR (cognit*) AND (impair* OR dysfunct* OR difficult* OR defect*))	
Total		1 paper
**EMBASE**		
Terms (actual search terms listed separately)	Balance training OR balance exercises	
	AND	
	(Dementia OR cognitive dysfunction OR Alzhiemer OR ((cognit*AND (impair* OR defect* OR difficult* OR dysfunc*)))	
Total		1 paper
Results		
Total		218 papers
Other sources		
Total after remove duplicate		1 paper

((("Dementia"[MeSH] OR "Cognitive Dysfunction"[MeSH] OR dementia*) OR ((cognit*) AND (((impair*) OR dysfunct*) OR difficult* OR defect*)))
AND ((balance AND (((impair*) OR dysfunct*) OR difficult* OR defect*)) OR accidental fall[Mesh]))
AND
((balance OR postural balance [Mesh] OR vestibular) AND (exercise [Mesh] OR training OR rehab* OR program))

Filters applied: RCT, humans, English, age 19+
